# Tipping points toward sustainability: The role of industrial ecology

**DOI:** 10.1111/jiec.70000

**Published:** 2025-03-20

**Authors:** Claudia R. Binder, Aristide Athanassiadis, David Bristow, Helmut Haberl, Christopher Kennedy

**Affiliations:** 1 Laboratory for Human-Environment Relations in Urban Systems, IIE, ENAC, Ecole Polytéchnique Fédéral de Lausanne, Lausanne, Switzerland; 2 Department of Civil Engineering, University of Victoria, Victoria, Canada; 3 Institute of Social Ecology, BOKU University, Vienna, Austria

**Keywords:** cities, climate change, industrial ecology, social metabolism, social science, tipping points

## Abstract

The discussion of tipping points (TPs) is getting increasing attention when addressing the climate and energy crisis. While industrial ecology (IE) has been playing a role in the climate and energy debates, it has so far fallen short of substantially adding to the research on TPs. This forum input highlights how the current contributions from IE have impacted the scientific community's understanding of TP. It highlights potential areas where IE engagement could be more prominent and points out avenues for achieving this. In particular, five fields of action seem pertinent: (i) engaging in theory building; (ii) closer collaboration with social sciences and economics; (iii) identifying and defining new indicators; (iv) making use of new modeling techniques; and (v) open science.

**Supplementary Information:**

The online version of this article (doi:10.1111/jiec.70000) contains supplementary material, which is available to authorized users.

## INTRODUCTION

Current climate, energy, and societal crises have shown that we need new tools for designing measures and strategies for achieving a more sustainable future. Scholars have claimed that an understanding of tipping points is crucial when trying to push societies toward sustainability transition pathways (Otto et al., 2020). Tipping points (TPs) are defined as “the point where a small intervention leads to a large and long-term consequence for the evolution of a complex system, profoundly altering its mode of operation” (Lenton and Latour, 2018; Lenton et al., 2022). When surpassing a TP, both the structure and the dynamics of the system change, while strongly reinforcing feedback loops emerge which can amplify a small change, accelerating the transition toward a new, difficult-to-revert, system state (Milkoreit et al., 2018).

In sustainability science, scholars sometimes distinguish between undesired and desired TPs, for example, based on international agreements such as the United Nations Framework Convention on Climate Change (UNFCCC) or the Convention on Biodiversity. Undesired TPs relate to changes that could or should be prevented, for example, surpassing planetary boundaries (Scheffer & Carpenter, 2003). Conversely, desired TPs relate to the possibility that nonlinearities in socio-ecological systems may also be used to successfully implement “social tipping interventions” that “activate contagious processes” of change toward climate stabilization (Otto et al., 2020, p. 1). To achieve such a transition, we need to push societal trajectories toward desirable tipping points, which could occur at different time and spatial scales. However, as argued in a recent conceptual paper, distinguishing “desirable” and “undesirable” pathways may be contested, given fundamental divergences in values and viewpoints between different social groups and regions (Brand et al., 2021). Biophysical stocks and flows underpin the delivery of services that are of key importance for social well-being (Brand-Correa et al., 2018). They are linked with practices, that is, everyday routines of dwelling, nourishing oneself, being mobile, acquiring competences, and so forth. Recognition of these close linkages underlies the complementary concepts of a “stock-flow-service” and a “stock-flow-practice” nexus (Haberl et al., 2021, 2023). They imply that tipping phenomena could result from disruptions of flows or destruction of stocks that trigger massive changes in services and practices (e.g., Singh et al., 2022), or vice versa result from success in altering dominant patterns of resource-intensive practices.

Industrial ecology is a scientific field that quantifies the flows and stocks of human activities and how they affect and are affected by the environment. It has provided a wealth of empirical data to characterize and model how anthropogenic activities mobilize and accumulate resource and pollution flows. However, TPs have so far not been an area of research. Most of the work in socio-metabolic research is based on methods that are poorly suited for addressing the challenge of analyzing tipping points. Many methods of substance, material, or energy flow analysis are either descriptive or rely on linear stock-flow models such as the MISO model (Wiedenhofer et al., 2024a). Such approaches could detect tipping points in hindsight but cannot be used for developing scenarios in which tipping effects occur due to nonlinear systemic feedback and interaction processes.

Industrial ecology does not yet have the capacity to simulate tipping points

An environmentally extended multi-regional input–output (EE-MRIO) analysis is highly useful to examine structural relationships, but is typically static and cannot, therefore, simulate nonlinear and tipping dynamics. The multi-scale integrated analysis of societal and ecosystem metabolism (MuSIASEM) approach aims to capture multiple scales and complex system thinking across dimensions (Giampietro et al., 2014), but has so far failed to generate algorithmic models capable of simulating the tipping dynamics of socio-ecological systems. Thus, there is a need to reflect on what role industrial ecology could play in analyzing, anticipating, and simulating tipping points. While the development of such models is beyond the scope of this article, we provide some discussions that may help with their design.

This paper addresses the following questions:

i. How can we characterize TPs from a flow and stock perspective? What additional information is required to understand tipping dynamics?
ii. How can we anticipate and model future negative and positive tipping points?
iii. What are the potential avenues for future research in industrial ecology?

## INSIGHTS ON TIPPING POINTS FROM A FLOW AND STOCK PERSPECTIVE

In the following section, we use examples from industrial ecology research that illustrate specific aspects of tipping points in socio-technical–ecological systems. We highlight the key messages from these examples in a box.

### The Wall Street Crash of 1929 as a tipping point in an energy transition

New research examining the Great Depression from a biophysical perspective shows that the infamous New York Stock Exchange crash of 1929 can be interpreted as a TP from the *Coal Age* to the *Oil Age* (Kennedy, 2023a, 2023b). Underlying the crash was a reduction of over 50% in oil prices and an announcement of oil supply certainty, after huge new oil fields were discovered in the US Southwest. The depression that followed entailed a painful transition in the American socio-technical regime (in the terminology of Hughes, 1987), when petroleum-based motor-vehicles surpassed coal-powered trains as the dominant US transportation system.

Tipping points from a material and energy perspective can induce fundamental transformation in a complex socio-economic system

**FIGURE 1:**
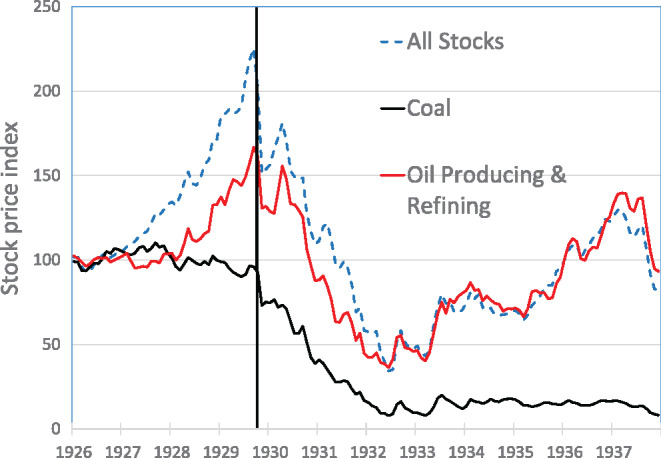
Price indexes for coal stocks, oil stocks, and all stocks on the New York Stock Exchange, 1926 to 1937 (monthly). The collapse in the coal index without recovery supports the notion that the Great Crash was a tipping point in an energy transition (figure 4 from Kennedy, 2024; data from: Cowles & Associates, 1938). Data can be found in the Supplementary Information Data For Figure 1: Supplementary Data _1_Great Crash.

Evidence that the Great Crash was a tipping point in an energy transition is provided, in part, by changes to the New York Stock Exchange (NYSE) indexes for coal and oil companies from 1926 to 1937 (Figure 1). The index of coal stock prices was relatively constant between 1926 and October 1929, before falling rapidly by approximately 90% by July 1932. Most notably, the coal index did not recover in the next 5 years. Behavior of the stock price index for oil production and refining companies was quite different; it followed the pattern of the aggregate stock market index, rising prior to the crash, sharply falling, and then recovering. By 1936, the stock price index for oil companies was back to its 1926 base value, but the price index for coal companies was still down by 85%.

Not all energy transitions necessarily involve major depressions (Kennedy, 2023b) and the circumstances of the current transition to renewables are different from those of 1929. Nonetheless, the case of the Great Crash demonstrates how a tipping point can be understood from a material and energy flow perspective, and how an energy transition entailed a fundamental transformation of a complex socio-economic system, including—amongst others—dominant practices of being mobile, as cars and airplanes became dominant mobility providers, replacing railways in the early decades of the 20th century.

### Tipping points and socio-metabolic regimes in Paris

In the case of Paris, there are several long-term urban metabolism studies that couple metabolic flow evolution with a description of infrastructure development and changes in legislation, as well as the interplay between stakeholders, including government officials, companies, and citizens. These studies provide the necessary empirical insights to characterize social tipping points for the flow of energy and water.

**FIGURE 2:**
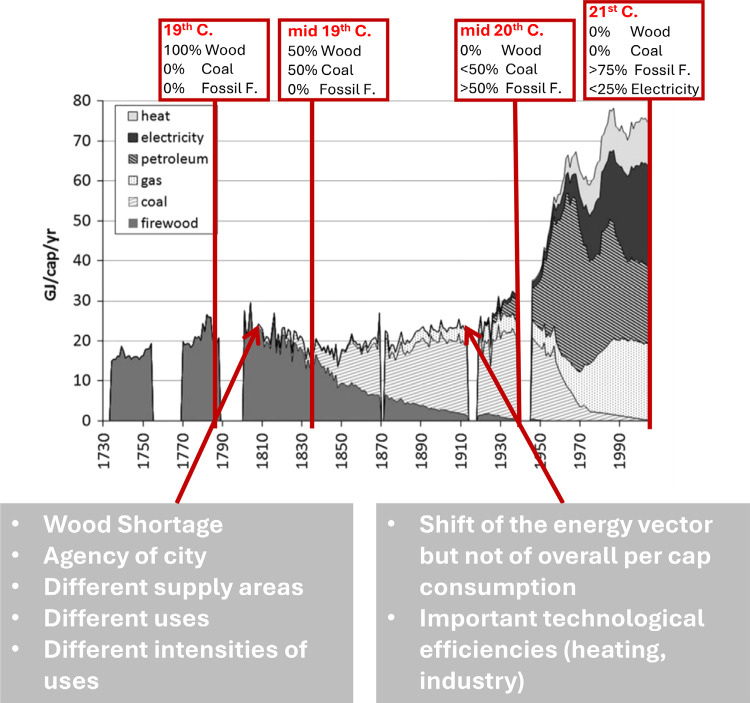
Total final energy consumption of Paris (GJ/cap/year) from 1730 to 2000. The mid-19th and mid-20th centuries show when the energy system passed from the predominance of one energy source in the energy mix to another, suggesting the presence of tipping points. Yet, the underlying factors and triggers behind the tipping points need to be found in articles and archives (graph adapted from Kim & Barles, 2012).

For both these flows, one or more events triggered a transition process which eventually led to a socio-metabolic lock-in effect, and which, in turn, led to a tipping point. In the case of energy, the trigger was a shortage of wood fuel, which led to the adoption of coal through a transformation of supply areas and the utilization of newly created railroads (see Figure 2; Kim & Barles, 2012).

In the case of water, the initial trigger was the challenge of both hygiene and sanitation (Barles, 2015). By developing the infrastructure to transport water in and out, the volumes flowing increased as well as their supply areas. Once these technological and infrastructural components were installed, and thanks to the role of engineers and doctors, who were responsible in part for decision-making, an infrastructural lock-in effect was implemented which opened and linearized Paris’ metabolism.

Tipping points in social-ecological–technical systems are interrelated and often a result of health or environmental triggers which launch a cascade of changes.

By developing long-term MFA studies, Barles and colleagues made possible the a posteriori identification of transitions between metabolic regimes. By complementing the MFA with institutional and infrastructural layers, it was possible to unveil the triggers and tipping points within these transition cycles. Finally, while these studies touched upon the average consumption per capita of energy and water flows over time, the relationship between infrastructural artifact and daily practices such as heating, hygiene, and nourishment, was less documented and could become a topic of further exploration.

### Tipping points related to phosphorous flows in Switzerland

The case of phosphorous (P) over time illustrates how environmental tipping, health prevention, and metabolic information triggered legislative changes which in turn impacted the metabolic system. Table Table 1 shows, in a simplified way, how a combination of several triggers led to or induced socio-political tipping and changes in legislation. We will exemplify some of the dynamics in two main episodes.

**TABLE 1:** The combination of several factors, metabolic insights, and environmental or human health issues lead to a TP and induced changes in the legislation system.

Crisis	Year	Trigger	Reactions/legislative system	Metabolic impact (P)
**Environmental health**	**1980s**	**Fish death**	● **Abolishment of P in detergents** ● **Development of “safety strips” from agricultural fields** ● **Oxygenation of lakes** ● **Chemical precipitation of P in wastewater treatment plants**	**Reduction of P concentration in lakes**
Environmental health	1990s	Groundwater contamination Metabolic insights	● Ordinance of direct payments (1993) ● Development of “safety strips” from agricultural fields ● Proof of ecological performance (1999)	Balanced N/P flows in agriculture Reduced increase of P stocks in soils
Human health	1990	Bovine spongiforme enzephalopathie (BSE) related health issues (humans and animals)	● Ban on meat and bone meal as animal feed (Ordinance on the Disposal of Animal By-Products [VTNP]) ● Specified risk material was fully removed (Ordinance on animal diseases [TSV])	Eradication of BSE Increase of P losses Reduction of circularity of P
Environmental health	2000	Soil contamination Metabolic insights	● Ban of direct use of sewage sludge in agriculture (2006) ● 10 years to reduce the input of sewage to soils to zero (Ordinance on Avoidance and Disposal of Waste [VVEA])	Increase of P losses Reduction of P circularity
**P as scarce resource**	**2007ff**	**Phosphorous peak** **Fear of lack of P supply** **Metabolic insights**	● **Technical recovery of P from municipal wastewater, sewage sludge and reutilization of meat and bone meal by 2026 (VVEA)**	

First, in Switzerland during the 1970s and 1980s, an increase in the P concentration in lakes took place, leading to algae bloom, a reduction in oxygen, and a decrease in the fish population (Knoepfel, 1995). The Federal Water Protection Act, implemented in 1972, mandated the treatment of industrial and household wastewater and the proportion of the population served by such plants rose from considerably less than 50% (in the 1970s) to more than 90% (in 1993). Additional measures in the agricultural sector were also implemented. Despite this, P concentration in some lakes continued to increase and scientists started to analyze the sources of the phosphorous inflow and the mechanism of the problems it caused (Knoepfel, 1995). Oxygenating lakes was very expensive and cantonal and national authorities became aware that additional measures needed to be taken to reduce the adverse effects of eutrophication (Knoepfel, 1995) “*We are convinced that the priority should be shifted to external measures: improvements in fertilising practices, restricted use of artificial fertilizers, adaptation of livestock holdings, improvements in the treatment of household wastewater, promotion of phosphate-free washing powders etc*.” (WWF statement, quoted in Knoepfel, 1995; p. 109). On August 7/8, 1984, a total of 325,000 fish died in Lake Sempach. This event led to an uproar in the population and a tipping dynamic that finally ended in several regulatory changes which supported the reduction of P inflows in lakes: (i) ban of P in detergents (ordinance on [Laundry] detergents, washing up liquids, and cleaning agents); (ii) integration of a fourth level for phosphate filtration in the waste water treatment plants (WWTP; Boller, 2013; Herman, 2009); and (iii) severe restrictions in the use of fertilizers in agriculture, particularly close to rivers and lakes (Knoepfel, 1995). Today, eutrophication mainly remains an issue in rivers and lakes surrounded by intensive stock farming and cultivation (Mehr et al., 2018).

Metabolic insights can provide a baseline for socio-political tipping, whereas crises can act as triggers or induce dynamics toward tipping.

Second, increasing phosphorus prices and supply uncertainty in the 2000s raised the question to what extent Switzerland could become more self-sufficient regarding its P supply. Indeed, according to Mehr et al. (2018), the total amount of P losses in waste management increased from 9000 t/year in 1989 to 13,300 t/year in 2015. Several studies (e.g., Binder et al., 2009; Binder & Jedelhauser, 2014; Lamprecht et al., 2011; Mehr et al., 2018) had identified the potential to increase circularity by extracting P from wastewater in the same order of magnitude as the current P imports via fertilizer. Based on these results and the supply uncertainty, the Ordinance on Avoidance and Disposal of Waste (VVEA) was introduced with the goal of “re-creating the P cycles between the waste sector and the agri-food system” (VVEA; Jedelhauser et al., 2018). Specifically, the VVEA asks for the technical recovery and recycling of P from municipal wastewater, sewage sludge, or sewage sludge ash and the re-utilization of P in meat and bone meal as of 2026. While it took almost 20 years from the first study to the mechanism's implementation, the final trigger was the perception of an acute P scarcity.

The example of P shows that crises related to environmental problems, human health (Bovine Spongiforme Enzephalopathie; BSE), or resource scarcity led to socio-political tipping and legislative reactions, and related changes in practices. For example, in the case of BSE, the use of meat and bone meal as animal feed for ruminants was banned, in 1990; specified risk material was fully removed from the feed chain in 1996; and animal byproducts were banned for fertilizer use in 2001 (FSVO, 2015; Mehr et al., 2018). In most cases (except BSE), MFAs or scientific metabolic studies created the basis for understanding the problems, developing solutions, and monitoring the outcomes. However, triggers were always needed to provoke a change in legislation and consequent effects on the metabolic system. Thus, metabolic studies can be essential for anticipating TPs and recasting the solutions for mitigating potential impacts.

## ANTICIPATING AND MODELING TIPPING POINTS

### Modeling tipping points in socio-ecological technical systems

To be able to capture TPs, models need to encapsulate nonlinearities and feedback mechanisms in systems over time that lead to fundamental structural changes in the modeled system. The mathematical and computational tools for establishing such models have advanced drastically in the last half-century (e.g., Fieguth, 2021; Gardiner, 2009; Strogatz, 2018; Thurner et al., 2018).

Techniques for establishing the appropriate form of a model in different application areas have also followed within the IE community. Particularly, current developments in LCA could provide relevant insights for analyzing the environmental impact of future trends in technologies. In addition, dynamic modeling of TPs could intersect with forward-looking work in LCA, whether using consequential techniques, considering future technologies or policies, tied to integrated assessment models (IAMs), or broadly using some form of transitional LCA (Mastrucci et al., 2024; Ventura, 2022).

Nevertheless, considering nonlinearities and feedback when modeling TPs in an industrial ecology context entails the following challenges:

First, there are **data challenges**. Industrial ecology datasets can often be limited to a few decades or less. Further, they may only capture annual changes, and finally, they may have a low signal-to-noise ratio depending on which dynamics are of interest. For instance, war may cause fluctuations in a socio-economic metabolism variable of interest that we might not have measured. The characteristics of many industrial ecology datasets can inhibit model fitting.

Higher resolution data and advancements in theory and integration with methods of tipping point detection and decision-making under uncertainty are needed

Second, there is both a need and opportunity for advancing **theory** that establishes support for different model choices. The appropriate models for capturing the dynamics of TP in socio-ecological systems are likely to require the integration of theory from other disciplines, including social sciences, engineering, planetary sciences, and complexity science—see Thurner et al. (2018). Many industrial ecology professionals and researchers are likely versed in one or more other disciplines. This represents a strength in this community for creating the necessary collaborations to model the types of tipping points discussed herein.

Third, a key challenge is the need to include **socio-economic dimensions** (e.g., actors, institutions, decision trees, practices, and provisioning systems and their services; Schaffartzik et al., 2021; Rao et al., 2019). Models capable of representing possible tipping dynamics (toward, e.g., sufficiency; Gough, 2023) in socio-ecological systems need to incorporate socio-economic and ecological phenomena at a similar level of complexity (e.g., spatial or temporal resolution, differentiation of subsystems or processes and feedbacks). Models that represent social dynamics via agent-based modeling (ABM) and integrate that with biophysical phenomena (e.g., climate extremes, changes in land-use intensity and farming practices, and use of construction materials as well as their consequences) could be a promising approach here (Egger et al., 2022; Egger et al., 2023; Knoeri et al, 2014; Mayer et al., 2022).

Finally, given the data and theory challenges, it is likely that, for the study of TPs in the near term, it will be possible to show that multiple models fit similarly well despite each having different tipping point characteristics and susceptibilities. Subsequently, it may be more appropriate to use **tipping point detection methods** in concert with efforts to identify appropriate models. This suggests that industrial ecology methods such as MISO and EE-MRIO can be used complementary to TP research outputs and expanded with for example Integrated Assessment Models (IAM). Furthermore, they will be most useful in practice with methods for decision-making under uncertainty.

### Early warning signals and establishing tipping point targets

The triggers and analyses presented suggest the question: what could be the early warning signs for future tipping points? Can we anticipate potential future tipping?

Early warning signals relate to different types of measures, indicating fast, frequent, and nonlinear changes with a high standard deviation in the variables measured (Scheffer et al., 2012). Further, the longer the time of recovery is following a perturbation, the more likely it is that the system is approaching a TP. This is supported in the literature on the modeling of nonlinear dynamic systems (Fieguth, 2021). Another early warning signal we could potentially identify is the delta of the growth rate. Exponential growth with a relatively short doubling time might indicate a rapid development, and thus, a rapid change of the system. Finally, frequent changes in events can be an early warning signal of an impending tipping point.

The potential for detecting TPs is also foundational to the concept of planetary boundaries (Steffen et al., 2015) of essential earth systems like the climate and ozone layer. Here, the boundaries are defined relative to zones of uncertainty around the tipping points in each system. The logic is that, for policy purposes, the planetary boundaries should be established conservatively outside the zone of uncertainty so as to reduce the risk of reaching one of the harmful earth-system tipping points. However, one needs to acknowledge that not all planetary boundaries are yet defined around tipping elements, for example, the land-use boundary is still rather arbitrary, demanding further research.

There are important links between planetary boundaries and industrial ecology TPS for sustainability. First, tipping socio-ecological systems toward sustainability is, in the aggregate, about keeping earth systems within planetary boundaries. Second, TPs for sustainability in socio-ecological systems should be established following a conservative approach concerning uncertainty similar to that used in the planetary boundaries approach. However, while the goal of the latter is to inhibit undesired tipping, that of socio-ecological sustainability targets is to ensure tipping.

## INDUSTRIAL ECOLOGY AND TIPPING POINTS: OPPORTUNITIES

This forum contribution reflected on how IE could contribute to the analyses of TPs in socio-ecological systems. Although IE has not been heavily involved in TP research, the examples presented show the relevance of engaging in, understanding, and modeling tipping points within the IE community. Particularly, we can draw on the insights and deep knowledge of how socio-metabolic profiles have fundamentally changed over time, for example, during the agrarian–industrial transition during which societies’ energy base shifted from biomass to fossil fuels (Fischer-Kowalski & Haberl, 2007). This transition had fundamental implications for society and the economy, as well as for resource-use patterns and sustainability problems. In the future, we need to either anticipate TPs in the social metabolism or speedily recognize their impacts. Let us mention only four of the several possible areas of concern, which we consider relevant to the IE community.

The low-carbon transition might be undertaken with greater control and consciousness than the transition to the Oil Age, as it is closely related to changes in the socio-metabolic profiles of our society. Whereas scholars have studied cascading and linkages between sectors such as energy production and storage, human settlements, and information feedback (Otto et al., 2020), with IE tools we could anticipate potential counterpoints such as dependency on critical minerals (IEA, 2023; IRP, 2023). Moreover, understanding the key role of changes in dominant practices may help in leveraging the large potentials identified in recent work (Creutzig et al, 2024; Hickel et al., 2022; Vogel et al., 2021) to provide societies with key services they need for their well-being at much lower levels of resource demand, and more circular resource flows, which can help in mitigating tradeoffs between clean-energy transitions and demand for critical raw materials.

### Infrastructure, lock-ins, and social metabolism

In the future development of carbon-neutral technologies (e.g., e-mobility), the role and interaction between infrastructure development, legislation, and adoption at the individual level is crucial (Sharpe & Lenton, 2021; Kekäle and Helo, 2014). Recent cross-country analyses have shown that size and spatial patterns of built structures (buildings, roads, and railways) co-determine per capita levels of energy, GHG emissions (Haberl et al., 2023), and materials use (Duro et al., 2024) about as strongly as GDP. These analyses statistically established that the effect is additional to the well-known correlation between resource use and GDP, and that built structures are much more important determinants of per capita resource use than other factors widely studied, for example, population density, climate related heating energy demand, or fuel prices. IE is already well positioned to understand systemic metabolic impacts of infrastructure change such as how energy systems affect life cycles of other processes. With IE tools and, in collaboration with social scientists and economists, IE can contribute to the simulation of the dynamics of these processes, including the material perspective, and comprising the embodied emissions (Mohareb & Kennedy, 2014).

Furthermore, the high cost and long life of infrastructure provides lock-ins and constrains development pathways. Helmrich et al. (2023) state that infrastructure can be viewed as an “agent against transformation” given that we can identify self-reinforcing feedbacks perpetuated by “individuals, organizations and institutions.” This lock-in can be further reinforced when purely technological alternatives are considered (Helmrich et al., 2023; Markolf et al., 2018).

### Cities and circularity

Given the key role of cities in climate transition, there is an urgent need to understand their dynamics from a coupled socio-ecological–technical perspective that accounts for nonlinearities (Bettencourt et al., 2007). With IE tools and linking up with social and political sciences, we could simulate the nonlinear dynamics of the urban environment, its potential development, and material consequences to finally derive information for knowledgeable decision-making (see Meirelles et al., 2021 for a first attempt). Indeed, TP has a role to play when simulating cities’ development as they “are largely built from the bottom up” and thus “if the essence or urban development is individual action, then a city can only be as smart as its citizens” (Batty, 2018, p. 177). Along these lines, during the last two decades, Batty and colleagues have studied the dynamics of urban territories using ABM and cellular automata (Batty, 2007, 2012) which could help modeling TPs at an urban level. More recently, these efforts were complemented with other methodological tools (scaling and power laws, Bettencourt, 2020, 2021; Roo & Rauws, 2012) to study the emergent properties of complex urban networks and build “an urban science.” Furthermore, technological development and emerging computational power has been used to develop urban observatories (https://newcastle.urbanobservatory.ac.uk/) and urban digital twins (Haken & Portugali, 2021) to measure, monitor, and simulate emergence and phase transitions in urban development.

Still, these analyses are relatively stock and flow agnostic and can also lack socio-political historical context which would help to explain how current practices were formed through metabolic lock-in effects resulting in a cascade of political, infrastructural, and other triggers. In the future, it would be relevant to cross-pollinate these two worlds by combining “urban science” methods with IE ones. For example, mapping the material stock and estimating material re-use in buildings is an increasingly active area of research and practice that could benefit from nonlinear dynamic stock models with economic, demographic, and technologic considerations. Further, the role of disruptions in causing changes in infrastructure patterns is an important topic requiring further consideration of potential long-term changes in urban metabolism (Magassy et al., 2023). Recently developed global maps of transport infrastructures (Wiedenhofer et al., 2024b) and buildings (Haberl et al., 2024) can provide valuable data for research in this field.

Another consideration for city researchers is to understand processes that lead cities to tipping points toward more sustainable directions. Examples include: London's implementation of congestion charging (Kennedy et al., 2005), Curitiba's revolutionary bus rapid transit system (Cervero, 1998) and rapid adoption of electric vehicles in Bergen, Oslo and other Norwegian cities (IEA, 2017).

### Supply-chain disruptions

Given current geopolitical turmoil, an open question is how supply-chain disruptions related to conflicts, wars, chance events (e.g., blocked shipping routes), pandemics, or climate change related extreme events will affect future socio-metabolic patterns? Up to what point are resource systems resilient toward such events and when will they result in tipping points where new patterns emerge? How do they affect efforts to work toward more sustainable resource use? IE could contribute to addressing these questions by linking up with complex system modelers, social scientists, economists, and foresight researchers. Recently developed big-data approaches could also be instrumental here (Reisch et al., 2022).

## NEXT STEPS

We have shown that TPs are important for understanding and for potentially pushing desired (stable) changes in the metabolic profiles of our societies. As material flows and stocks are the expression of such profiles, they could provide indicators for anticipating tipping points. We can see the role of metabolic research in:

- **Understanding,** through modeling and empirical research, the mechanisms of TPs.
- **Envisioning sustainable metabolic states** through scenario modeling, stakeholder engagement and systemic analyses.
- **Preparing** for TPs by developing metabolic evidence which, together with social models and systemic perspectives, allows for knowledgeable decision-making.
- **Informing** stakeholders to navigate the climate and energy transitions through knowledge on the mechanisms of TPs.
- **Detecting** early warning signals.
- **Governing** TPs, by understanding the needs of the citizens and making tradeoffs explicit.
- **Monitoring** the transition by developing appropriate indicators and measures to follow the pathway and anticipate disruptions.

How can IE move toward these contributions? We consider five aspects to be relevant: (i) engaging in theory building; (ii) closer collaboration with social sciences and economics; (iii) identifying and defining new indicators; (iv) making use of new modeling techniques: and (v) open science.

### Theory building

#### How can we enhance our theoretical perspective by including the notion of tipping?

One possibility to enhance theory building is to develop a link with the resilience community. The resilience-focused tradition of socio-ecological systems research deals with nonlinearity and complex systems thinking at a conceptual level. Many case studies within this research tradition have grappled with the resilience of specific resource systems, including governance systems and key actors (Folke et al., 2021); guided by conceptual frameworks such as the “lazy eight” metaphor (Gunderson & Holling, 2002). So far, few, if any, algorithmic models have been developed to assess such phenomena (potentially including tipping points); for a recent attempt see Castell and Schrenk (2020) and Meerow and Newell (2015). By bridging these research traditions, an innovative research field could emerge that could develop methods and models capable of dealing with nonlinear dynamics of networked, complex systems characterized by phenomena such as percolation (phase transitions) or other kinds of system reorganization leading to rapid, irreversible change.

### Closer collaboration with social sciences and economics

#### How can we orchestrate social tipping and social metabolism?

The literature on social tipping has increased during recent years (see Eker et al., 2023b; Otto et al., 2020), though neglecting the socio-metabolic perspective. Thus, the industrial ecology community could benefit from behavioral, policy science, as well as science and technology studies to identify drivers and barriers to change and the metabolic insights required for inducing these changes. This would be particularly useful when studying the effects of (i) resource constraints, for example, Paris (Barles & Kim, 2012); (ii) the shift to a qualitatively new resource base, for example, the switch to coal, then oil, and later natural gas as fuels for industrialization (Fischer-Kowalski & Haberl, 2007; Sieferle, 2001; Sieferle et al., 2006); (iii) environmental health (Mehr et al., 2018); (iv) social movements, such as Fridays for Future (Fritz et al., 2023); or (v) economic crisis (Kennedy, 2023a, 2023b).

Industrial ecology needs to open up to other disciplines to take full leverage of its capacity to contribute to tipping point research

Finally, one important notion for positive tipping points is that they are desired or even intentional. However, although we plan for a TP, even price signals might not lead to the desired scenario. Collaboration with social sciences might elucidate reasons such as resistance, vested interests, and heterogeneous consumers who might not adopt new behaviors and technologies concomitantly.

### Identify and define TP indicators

#### What are adequate indicators to be monitored to provide early recognition of tipping points in socio-metabolic systems?

Increasing theoretical breadth by opening up to social science and economics brings the potential to innovate and define new indicators. These indicators will contribute to identifying and anticipating TPs as well as understanding potential cascading dynamics. It is particularly relevant to leverage the historical metabolic data, as well as emerging data sets, and combine them with relevant socio-economic data.

### Making use of new modeling technologies

#### How can we further leverage big data and simulation techniques to simulate and anticipate TPs?

One option for developing such models relates to the use of novel computational technologies. Current big-data approaches to complexity science work with highly granular data, for example, all transactions between firms in a national economy (see Diem et al., 2022). Such models are not yet linked with biophysical data but doing so (similarly to how material flows are linked with intersectoral flows in EE-MRIO models) would open up an entirely new strategy to build “mechanistic,” nonlinear socio-metabolic models which could help analyze tipping dynamics.

### Open science

#### How can we better foster and make use of open science principles?

Development of indicators, simulation models, and interdisciplinary collaboration requires the fostering of a culture of open science and open data practices, particularly in regard to time series data as well as code. It is essential to build and cultivate the current efforts in our community (see Pauliuk, 2019) aimed at making data reproducible and comparable.

We are convinced that IE can make significant contributions to the early recognition of undesired tipping points within metabolic profiles and move toward anticipating desired tipping points in collaboration with other disciplines. We look forward to further discussions.

## Supplementary Information

Data for Figure 1: Price indexes for coal.

Supporting info item

## Data Availability

For Figure 1, the data are provided in Supplementary Data 1_Great Crash. For Figure 2, we obtained the rights to reuse the image given we acknowledge the source. See https://link.springer.com/article/10.1007/s10113-011-0275-0#rightslink. In that article the original dataset was not provided.
