# Supplementary material for: Tipping points toward sustainability: The role of industrial ecology
Source: J Ind Ecol. 2025 Mar 20;29(3):622–33. doi: 10.1111/jiec.70000 (PMC13111511; doi:10.1111/jiec.70000)
Supplement: Supplementary file 1 — Supporting info item [file 44498_2025_2903002_MOESM1_ESM.pdf]

2023 Journal of Industrial Ecology – www.wileyonlinelibrary.com/journal/jie

**SUPPORTING INFORMATION FOR:**

Binder et al  (2024). Tipping points towards sustainability: the ro
*Journal of Industrial Ecology.*

**Supplementary Information 1:  Data for figures**

Tab 1 contains the data set for  Figure 1

ɔle of Industrial Ecology.

| | 1926 | | | | | | |
|---|---|---|---|---|---|---|---|
| | Jan | Feb | Mar | Apr | May | Jun | July |
| All Stocks | 101.8 | 101.8 | 95.8 | 92.9 | 93.2 | 97.2 | 100 |
| Coal | 99.1 | 98.8 | 93.7 | 93.6 | 95.8 | 98.2 | 97.8 |
| Oil Producing & Refining | 102.6 | 101 | 98.7 | 96.2 | 97.9 | 100.3 | 101.5 |

Soure: Cowles, A. and Associates (1938) Common-stock indexes 1871-1937, Principia Press Inc. Bloomir

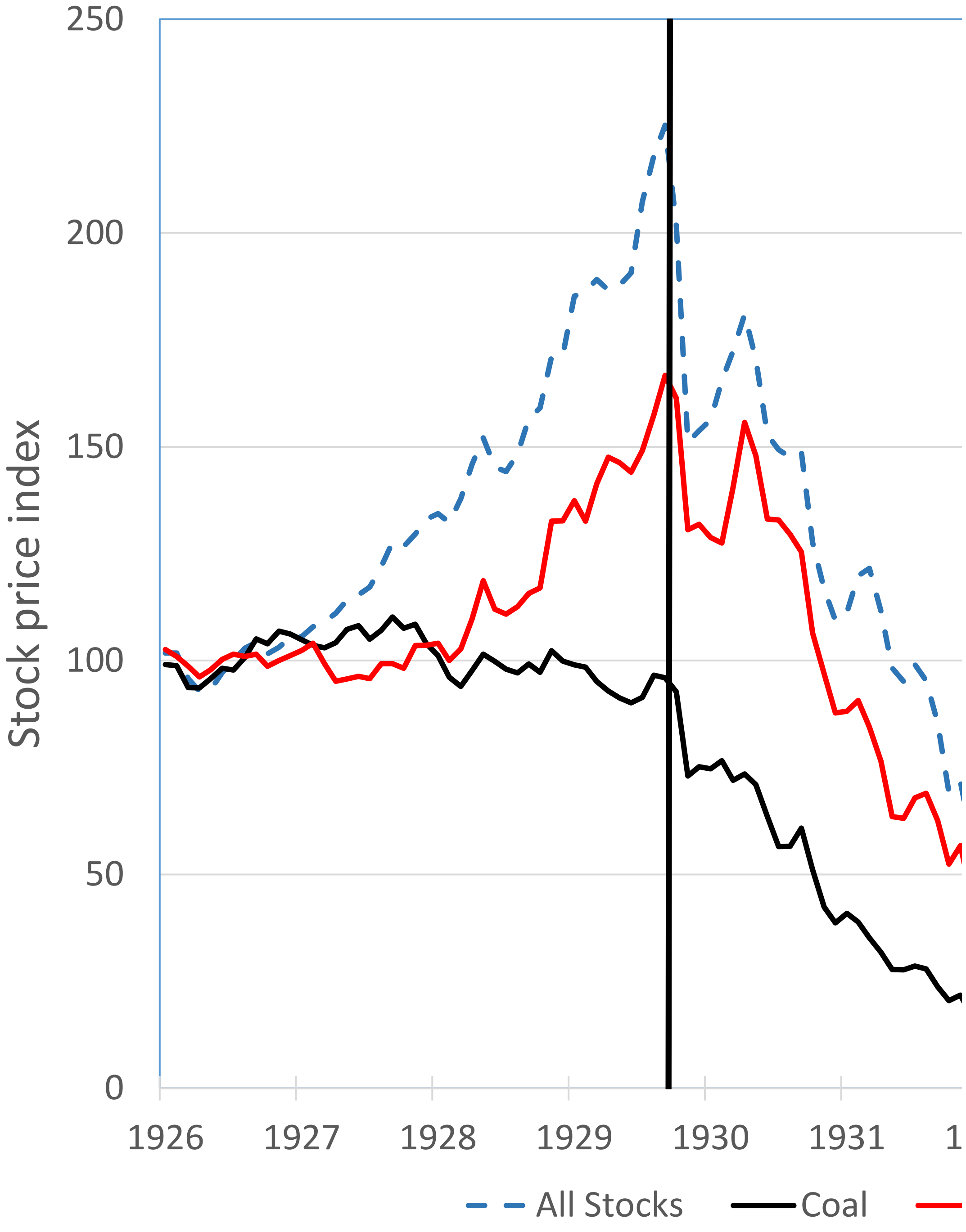

| | | | | | 1927 | | | | |
|---|---|---|---|---|---|---|---|---|---|
| Aug | Sep | Oct | Nov | Dec | Jan | Feb | Mar | Apr | May |
| 102.9 | 104.3 | 101.6 | 103.1 | 105.4 | 105.6 | 107.9 | 109.1 | 111.1 | 114.2 |
| 100.8 | 105.1 | 103.9 | 106.9 | 106.2 | 104.9 | 103.6 | 103 | 104.2 | 107.3 |
| 101 | 101.5 | 98.7 | 100 | 101.2 | 102.4 | 104.1 | 99.3 | 95.2 | 95.7 |

ıgton, Indiana.

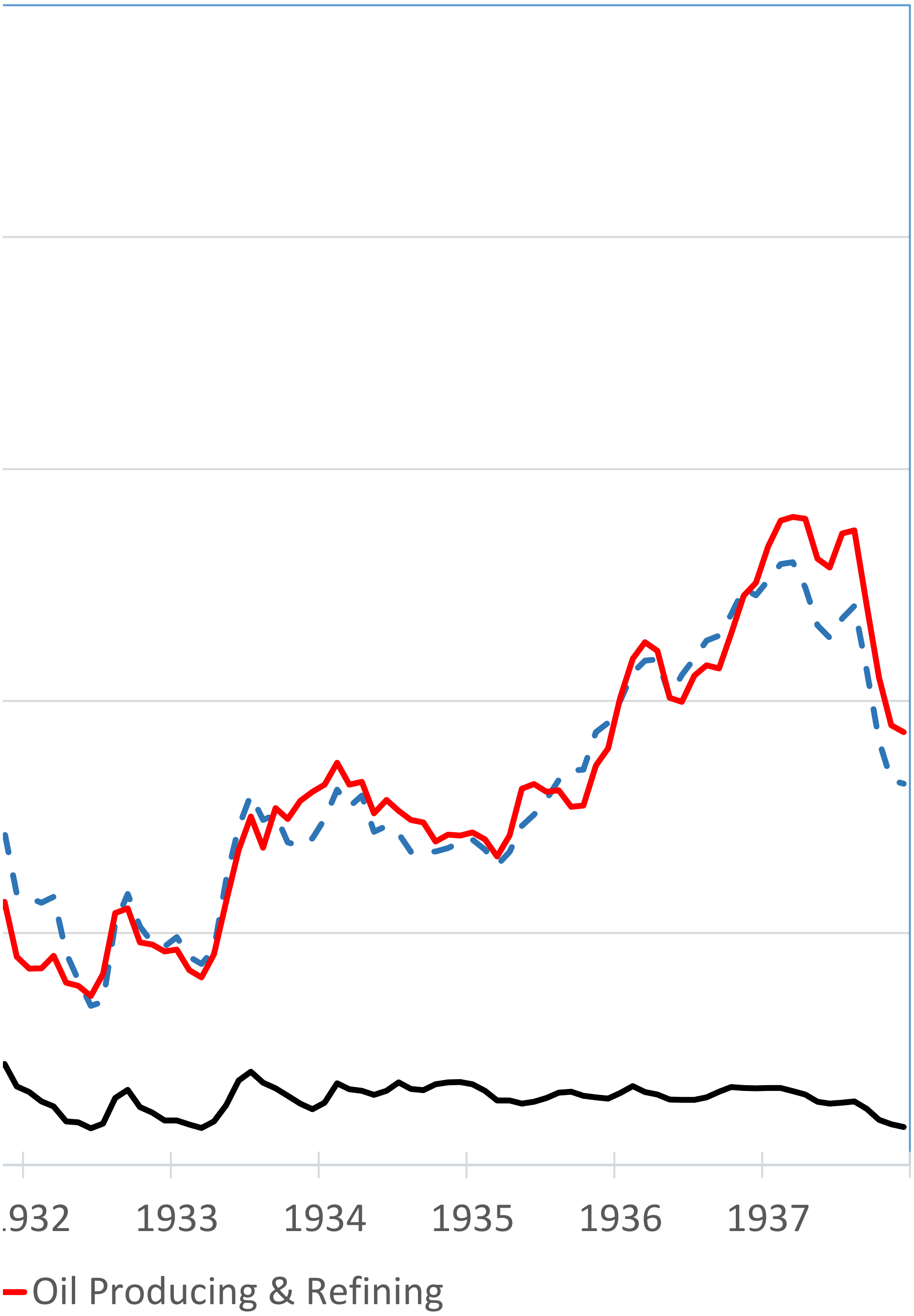

| | | | | | | | | | |
|---|---|---|---|---|---|---|---|---|---|
| | | | | | | | 1928 | | |
| Jun | July | Aug | Sep | Oct | Nov | Dec | Jan | Feb | Mar |
| 115.4 | 117.2 | 122 | 127.7 | 126.7 | 129.6 | 133.1 | 134.4 | 132.3 | 137.9 |
| 108.2 | 105 | 107.1 | 110.2 | 107.6 | 108.5 | 103.9 | 101.2 | 96.1 | 94 |
| 96.3 | 95.8 | 99.3 | 99.3 | 98.2 | 103.5 | 103.6 | 104.1 | 100 | 102.7 |

| | | | | | | | | | 1929 |
|---|---|---|---|---|---|---|---|---|---|
| Apr | May | Jun | July | Aug | Sep | Oct | Nov | Dec | Jan |
| 145.9 | 152.1 | 145.3 | 144.2 | 148.3 | 156.6 | 159.1 | 171.1 | 171.4 | 185.2 |
| 97.7 | 101.5 | 99.8 | 98 | 97.1 | 99.2 | 97.3 | 102.3 | 99.9 | 99 |
| 109.7 | 118.7 | 112 | 110.9 | 112.6 | 115.7 | 117 | 132.6 | 132.7 | 137.4 |

| Feb | Mar | Apr | May | Jun | July | Aug | Sep | Oct | Nov |
| --- | --- | --- | --- | --- | --- | --- | --- | --- | --- |
| 186.5 | 189.1 | 186.6 | 187.8 | 190.7 | 207.3 | 218.1 | 225.2 | 201.7 | 151.1 |
| 98.5 | 95.1 | 92.9 | 91.3 | 90.1 | 91.4 | 96.6 | 96 | 92.7 | 73 |
| 132.6 | 141.4 | 147.6 | 146.3 | 144.1 | 149.1 | 157.5 | 166.7 | 161.4 | 130.6 |

| | | | | | | | | | |
|---|---|---|---|---|---|---|---|---|---|
| | 1930 | | | | | | | | |
| Dec | Jan | Feb | Mar | Apr | May | Jun | July | Aug | Sep |
| 153.8 | 156.3 | 165.5 | 172.4 | 181 | 170.5 | 152.8 | 149.3 | 147.6 | 148.8 |
| 75.2 | 74.7 | 76.6 | 72 | 73.5 | 71 | 63.5 | 56.5 | 56.6 | 60.8 |
| 131.9 | 128.8 | 127.5 | 140.6 | 155.7 | 147.9 | 133.1 | 132.9 | 129.5 | 125.4 |

| | | | | | | | | | |
|---|---|---|---|---|---|---|---|---|---|
| | | | 1931 | | | | | | |
| Oct | Nov | Dec | Jan | Feb | Mar | Apr | May | Jun | July |
| 127.6 | 116.7 | 109.4 | 111.3 | 119.8 | 121.6 | 111.6 | 98.3 | 95.1 | 99.1 |
| 51.1 | 42.4 | 38.7 | 40.9 | 38.9 | 35.2 | 31.8 | 27.8 | 27.7 | 28.6 |
| 106.4 | 96.9 | 87.8 | 88.2 | 90.7 | 84.5 | 76.5 | 63.5 | 63.1 | 67.9 |

| | | | | | | | | | |
|---|---|---|---|---|---|---|---|---|---|
| | | | | | 1932 | | | | |
| Aug | Sep | Oct | Nov | Dec | Jan | Feb | Mar | Apr | May |
| 95.3 | 85.4 | 69.2 | 71.7 | 58.4 | 57.5 | 56.5 | 57.8 | 45.7 | 39.8 |
| 27.9 | 23.7 | 20.5 | 21.8 | 16.9 | 15.7 | 13.7 | 12.6 | 9.4 | 9.2 |
| 69 | 62.6 | 52.4 | 56.7 | 44.9 | 42.3 | 42.4 | 45.1 | 39.3 | 38.6 |

| | | | | | | | | | |
|---|---|---|---|---|---|---|---|---|---|
| | | | | | | | 1933 | | |
| Jun | July | Aug | Sep | Oct | Nov | Dec | Jan | Feb | Mar |
| 34.3 | 35.2 | 52.1 | 58.4 | 51.4 | 47.9 | 47.1 | 49.1 | 44.9 | 43.3 |
| 7.9 | 8.9 | 14.4 | 16.2 | 12.5 | 11.3 | 9.6 | 9.6 | 8.7 | 8 |
| 36.4 | 41.2 | 54.3 | 55.3 | 48 | 47.5 | 46 | 46.4 | 42 | 40.4 |

| | | | | | | | | | 1934 |
|---|---|---|---|---|---|---|---|---|---|
| Apr | May | Jun | July | Aug | Sep | Oct | Nov | Dec | Jan |
| 46.5 | 61.5 | 72.8 | 79.8 | 74.4 | 75.5 | 69.5 | 68.8 | 70.4 | 74.6 |
| 9.4 | 12.9 | 18.2 | 20.1 | 17.7 | 16.5 | 14.9 | 13.2 | 12 | 13.4 |
| 45.4 | 56.8 | 67.9 | 75.1 | 68.4 | 76.9 | 74.6 | 78.5 | 80.4 | 82 |

| Feb | Mar | Apr | May | Jun | July | Aug | Sep | Oct | Nov |
|---|---|---|---|---|---|---|---|---|---|
| 80.9 | 77.2 | 79.6 | 71.8 | 73.1 | 71.4 | 67.5 | 67.4 | 67.6 | 68.3 |
| 17.6 | 16.3 | 16 | 15.1 | 16 | 17.8 | 16.4 | 16.1 | 17.4 | 17.8 |
| 86.7 | 82 | 82.6 | 75.8 | 78.7 | 76.3 | 74.4 | 73.8 | 69.7 | 71.2 |

| | 1935 | | | | | | | | |
|---|---|---|---|---|---|---|---|---|---|
| Dec | Jan | Feb | Mar | Apr | May | Jun | July | Aug | Sep |
| 69.6 | 70.1 | 68 | 64.6 | 67.5 | 73.1 | 75.5 | 78.8 | 83 | 85 |
| 17.9 | 17.4 | 16 | 13.9 | 13.9 | 13.2 | 13.6 | 14.4 | 15.6 | 15.8 |
| 71 | 71.7 | 70.2 | 66.5 | 71.1 | 81.1 | 82.1 | 80.4 | 80.8 | 77.2 |

| | | | | | | | | | |
|---|---|---|---|---|---|---|---|---|---|
| | | | 1936 | | | | | | |
| Oct | Nov | Dec | Jan | Feb | Mar | Apr | May | Jun | July |
| 85.2 | 93.3 | 95.3 | 100.1 | 106.1 | 108.7 | 109 | 101 | 105.6 | 109.2 |
| 14.9 | 14.6 | 14.3 | 15.5 | 17 | 15.7 | 15.2 | 14.1 | 14 | 14 |
| 77.5 | 86.1 | 89.8 | 100.8 | 109.1 | 112.7 | 110.8 | 100.7 | 99.8 | 105.5 |

| | | | | | 1937 | | | | |
|---|---|---|---|---|---|---|---|---|---|
| Aug | Sep | Oct | Nov | Dec | Jan | Feb | Mar | Apr | May |
| 113 | 114.1 | 118.7 | 124.2 | 122.8 | 126 | 129.5 | 129.9 | 124.5 | 116.3 |
| 14.6 | 15.8 | 16.8 | 16.6 | 16.5 | 16.6 | 16.6 | 15.9 | 15.2 | 13.6 |
| 107.7 | 107 | 114.6 | 122.7 | 125.5 | 133.3 | 138.9 | 139.7 | 139.3 | 130.7 |

| Jun | July | Aug | Sep | Oct | Nov | Dec |
|---|---|---|---|---|---|---|
| 113.6 | 117.8 | 120.5 | 106.4 | 91.4 | 82.9 | 82.2 |
| 13.2 | 13.4 | 13.7 | 12.1 | 9.7 | 8.8 | 8.2 |
| 128.8 | 136.1 | 136.8 | 120.4 | 105 | 94.8 | 93.3 |
